# Acteoside and ursolic acid synergistically protects H_2_O_2_-induced neurotrosis by regulation of AKT/mTOR signalling: from network pharmacology to experimental validation

**DOI:** 10.1080/13880209.2022.2098344

**Published:** 2022-09-14

**Authors:** Yan-Jie Qu, Min-Rui Ding, Chao Gu, Li-Min Zhang, Rong-Rong Zhen, Jin-Fang Chen, Bing Hu, Hong-Mei An

**Affiliations:** a Department of Neurology, Longhua Hospital, Shanghai University of Traditional Chinese Medicine, Shanghai, China; b Department of Traditional Chinese Medicine, Ruijin Hospital, Shanghai Jiao Tong University School of Medicine, Shanghai, China; c Department of Oncology, Institute of Traditional Chinese Medicine in Oncology, Longhua Hospital, Shanghai University of Traditional Chinese Medicine, Shanghai, China; d Department of Science & Technology, Longhua Hospital, Shanghai University of Traditional Chinese Medicine, Shanghai, China

**Keywords:** Neuroprotection, apoptosis, mTOR-dependent autophagy, *Rehmannia glutinosa* (Gaetn.) Libosch. ex Fisch. et Mey., *Cornus officinalis* Sieb. et Zucc

## Abstract

**Context:**

Ursolic acid (UA) and acteoside (ATS) are important active components that have been used to treat Alzheimer’s disease (AD) because of their neuroprotective effects, but the exact mechanism is still unclear.

**Objective:**

Network pharmacology was used to explore the mechanism of UA + ATS in treating AD, and cell experiments were used to verify the mechanism.

**Materials and methods:**

UA + ATS targets and AD-related genes were retrieved from TCMSP, STITCH, SwissTargetPrediction, GeneCards, DisGeNET and GEO. Key targets were obtained by constructing protein interaction network through STRING. The neuroprotective effects of UA + ATS were verified in H_2_O_2_-treated PC12 cells. The subsequent experiments were divided into Normal, Model (H_2_O_2_ pre-treatment for 4 h), Control (H_2_O_2_+ solvent pre-treatment), UA (5 μM), ATS (40 μM), UA (5 μM) + ATS (40 μM). Then apoptosis, mitochondrial membrane potential, caspase-3 activity, ATG5, Beclin-1 protein expression and Akt, mTOR phosphorylation levels were detected.

**Results:**

The key targets of UA + ATS-AD network were mainly enriched in Akt/mTOR pathway. Cell experiments showed that UA (ED_50_: 5 μM) + ATS (ED_50_: 40 μM) could protect H_2_O_2_-induced (IC_50_: 250 μM) nerve damage by enhancing cells viability, combating apoptosis, restoring MMP, reducing the activation of caspase-3, lessening the phosphorylation of Akt and mTOR, and increasing the expression of ATG5 and Beclin-1.

**Conclusions:**

ATS and UA regulates multiple targets, bioprocesses and signal pathways against AD pathogenesis. ATS and UA synergistically protects H_2_O_2_-induced neurotrosis by regulation of AKT/mTOR signalling.

## Introduction

Alzheimer’s disease (AD) is a common neurodegenerative disease, characterized by memory loss, a progressive decline in cognitive ability, and difficulties with daily activities until an individual loses autonomy completely (DeTure and Dickson 2019). It is estimated that the number of AD patients will reach 152 million by 2050 (Xie et al. 2020). The main pathological changes of AD are senile plaques with amyloid-β protein as the core, neurofibrillary tangles formed by abnormal aggregation of hyperphosphorylated Tau protein, and loss of hippocampal neurons (Mohamed et al. 2016). Both amyloid-β and Tau can induce oxidative stress and contribute to AD pathogenesis (Zhao and Zhao 2013; Cheignon et al. 2018). So far, anti-AD drugs include cholinesterase inhibitors, NMDA receptor antagonists, β-site amyloid precursor protein cutting enzyme 1(BACE1) inhibitors, and some protein kinase inhibitors, which can only delay the progression of the disease and cannot cure AD.

Studies have shown that Chinese herbal compounds play active roles in the prevention and treatment of AD (Li et al. 2016). Liu-Wei-Di-Huang Decoction (Wang et al. 2017) and Di-Huang-Yi-Zhi (An et al. 2017) are essential traditional Chinese medicine (TCM) prescriptions against AD. *Rehmannia glutinosa* (Gaetn.) Libosch. ex Fisch. et Mey. (Scrophulariaceae) (Shu-Di-Huang) and *Cornus officinalis* Sieb. et Zucc. (Cornaceae) (Shan-Zhu-Yu) are important components of these. Studies have shown that *C. officinalis* can induce anti-AD effects by inhibiting Aβ_1-42_-induced apoptosis and inflammatory response, Tau hyperphosphorylation and aggregation (Ma et al. 2020), and cholinesterase and BACE1 activity. *R. glutinosa* improves cognitive function by alleviating the decrease of cholinergic immune reactivity and inflammatory response in the hippocampus (Lee et al. 2011) and upregulating the expression of glial cell lines-derived neurotrophic factor (GDNF) in astrocytes (Yu et al. 2006). Ursolic acid (UA), a pentacyclic triterpenoid compound, is one of the essential active components of *C. officinalis* Sieb. et Zucc. (Cornaceae). It has been extensively studied in tumours, diabetes, liver disease, etc., and has antitumor, antioxidant, anti-inflammatory, hypoglycaemic, anti-hepatic fibrosis, and other effects (Hussain et al. 2017; Gan et al. 2018). In recent years, there have been many reports on the neuroprotective effect of UA against neurodegenerative diseases, ischaemic cerebrovascular diseases and spinal cord injury. As an iridoid glycoside compound, acteoside (ATS) is one of the essential active components of *R.* *glutinosa* (Gaetn.) Libosch. ex Fisch. et Mey. (Scrophulariaceae). It has many physiological functions such as anti-inflammation, neuroprotection, immunomodulation, antitumor and wound healing effects; of these, the neuroprotective effect against neurodegenerative diseases has been widely reported.

Network pharmacology has been an emerging subject in recent years. Network pharmacology can help to study the specific targets of active ingredients in traditional Chinese medicines and define their functions in the context of molecular networks (Luo et al. 2020). Molecular docking is a theoretical simulation method for predicting intermolecular binding patterns and affinity, predicting drug target interactions with high efficiency and low cost (Wu et al. 2018). Based on previous studies, we analyzed the key targets and signal pathways of UA and ATS on AD by using network pharmacology and molecular docking methods, and verified that the combination of UA and ATS can inhibit the apoptosis of PC12 cells induced by H_2_O_2_ through cell experiments and the mechanism may be related to the regulation of Akt/mTOR signalling.

## Materials and methods

### Collection of compound-related targets and AD-related targets

The targets of UA and ATS were retrieved and predicted by TCMSP (http://lsp.nwu.edu.cn/browse.php), STITCH (http://stitch.embl.de/) (Szklarczyk et al. 2016) and SwissTargetPrediction (http://www.swisstargetprediction.ch/) (Daina et al. 2019) according to chemical similarity and pharmacophore model. Online Mendelian Inheritance in Man (OMIM, https://omim.org/) (Amberger et al. 2019), DisGeNET (http://www.disgenet.org/) (Pinero et al. 2017), GeneCards (https://www.genecards.org/) (Safran et al. 2010) and The National Centre for Biotechnology Information (NCBI) Gene Expression Omnibus (GEO) database (http://www.ncbi.nlm.nih.gov/geo) were used to screen AD-related genes to establish AD target data set. The relevant screening criteria and datasets selected in the GEO were previously described (Qu et al. 2020). All targets were unified as gene names by searching the UniprotKB database (https://www.uniprot.org/) or STRING database (https://string-db.org/) and selecting the species ‘Homo sapiens.’

### Network construction and central network topological analysis

The protein-protein network was constructed through the STRING database, and the target with a confidence score ≥ 0.7 was selected. The following network construction and topology analysis were carried out using Cytoscape 3.6.0 software: ①The target network of UA + ATS；②The AD-related target network; ③The UA + ATS target-AD target interaction network; ④The networks of shared targets between UA + ATS and AD targets. Topology parameters, such as degree centrality (DC), betweenness centrality (BC), and closeness centrality (CC), were used to evaluate the central properties of nodes in a network. In the UA + ATS target-AD target interaction network, the parameter settings of DC ≥ 3 × median DC, BC ≥ median BC, and CC ≥ median CC were used to screen the key targets of UA + ATS.

### Gene ontology (GO) and KEGG pathway enrichment analysis

The key targets of UA and ATS that act on AD were imported into DAVID 6.8 (https://david.ncifcrf.gov/) (Huang da et al. 2009), respectively. The procedure was consistent with that in our previously published network pharmacological study. GO and pathway were plotted by Bioinformatics (http://www.bioinformatics.com.cn/), an online platform for data analysis and visualization.

### Validation of binding capacity between active ingredients and key targets by molecular docking

Mol2 files were downloaded from the ZINC database (http://zinc15.docking.org) (Doytchinova et al. 2018) and converted to PDB format using PyMol software. Auto Dock 1.5.6 software was used for hydrogenation, charge balance, and other operations. The 3D structure of related proteins was downloaded from the PDB database (https://www.rcsb.org/), and the process of dehydrating and hydrogenation of proteins was carried out with PyMol software. Subsequently, Auto Dock 1.5.6 software converted the receptor and ligand into pdbqt format for molecular docking. Finally, PyMol software was used for plotting.

### Materials

UA (Lot No.110742-201823) and ATS (Lot No.111530-201914) were purchased from China National Institute for Food and Drug Control (Beijing, China). RPMI-1640 medium was purchased from HyClone (Logan, UT, USA). Foetal bovine serum (FBS), penicillin-streptomycin, and trypsin were purchased from Gibco (Grand Island, NY, USA). Cell counting kit-8 (CCK-8), Caspase-3 kit, and mitochondrial membrane potential (MMP) assay kit with JC-1 were purchased from Beyotime Biotechnology (Haimen, Jiangsu, China). The apoptosis detection kit was purchased from BD Biosciences (San Jose, CA, USA). Antibodies against GAPDH, Akt, p-Akt, mTOR, p-mTOR were purchased from Bioworld (St. Louis Park, MN, USA).

### Cell culture

Low-differentiated PC12 cells were purchased from The Cell Bank of Type Culture Collection of the Chinese Academy of Sciences (Shanghai, China). PC12 cells were cultured in RPMI-1640 medium containing 10% FBS and 1% penicillin-streptomycin, placed in an incubator with 5% CO_2_ at 37 °C, and logarithmic phase cells with good growth status were taken for subsequent experiments.

### Cell viability

PC12 cells were sewed on a 96-well plate (6 × 10^4^/well) and cultured for 24 h, then treated with H_2_O_2_ for 4 h, and the cell viability was evaluated by a CCK-8 kit according to the manufacturer’s manual. In another experiment, PC12 cells were incubated with UA and, or ATS for 24 h, and then treated with 250 μM H_2_O_2_ for 4 h, and the cell viability was evaluated by a CCK-8 kit. Cell survival (%) = (experimental OD value/control OD value) × 100%.

### Cell apoptosis detected by Hoechst 33258 and Annexin V-FITC/PI staining

PC12 cells were inoculated on 24-well plates at 1 × 10^5^/well and culture for 24 h, and then incubated with UA (5 μM) and, or ATS (40 μM) for 24 h, and then treated with 250 μM H_2_O_2_ for 4 h, washed with PBS, stained with Hochest33258 for 15 min, and observed and photographed under a fluorescence microscope. For apoptosis quantification, PC12 cells were taken on 6-well plates at 2 × 10^5^/well and culture for 24 h, and then incubated with UA (5 μM) and, or ATS (40 μM) for 24 h, and then treated with 250 μM H_2_O_2_ for 4 h. The cells were collected, resuspended with binding buffer, stained with 5 μL PI for 15 min and 5 μL Annexin V-FITC for 5 min. The apoptotic cells were analyzed by flow cytometry.

### Caspase-3 activity assay

PC12 cells were treated as described above and lysed, quantified. The activity of caspase-3 was detected according to the manufacturer’s protocol.

### JC-1 staining

PC12 cells were inoculated on 24-well plates at 1 × 10^5^/well, and treated as above. The cells were stained with JC-1 solution for 30 min at 37 °C and observed under a fluorescence microscope.

### Western blot

Treated PC12 cells were collected, washed and centrifuged, lysed by RIPA buffer, and quantified using the BCA kit. After the SDS-PAGE gel was prepared, the gel plate was placed in the electrophoresis tank, and equal quality samples were added to each well for electrophoresis for 100–120 min at 120 V. The protein in the gel was transferred to the PDVF membrane by the current of 260 mA for 80–100 min. The membranes were blocked with 3% BSA for 1 h, incubated with antibodies against Akt, p-Akt, mTOR, p-mTOR, or GAPDH (1:1000) at 4 °C overnight. The blots were washed with TBST and incubated with a secondary antibody (1:10000) at 37 °C for 1 h. The blots were visualized by the ECL reagent. Protein expressions were quantified by Image J software.

### Statistical analysis

IBM SPSS 21.0 software was used for statistical analysis. All data were expressed as mean ± SD. Comparison between two groups was performed by independent sample *t*-test (normal distribution) or rank-sum test (non-normal distribution). *p* < 0.05 was considered statistically significant.

## Results

### Targets of UA and ATS

**Figure 1.**
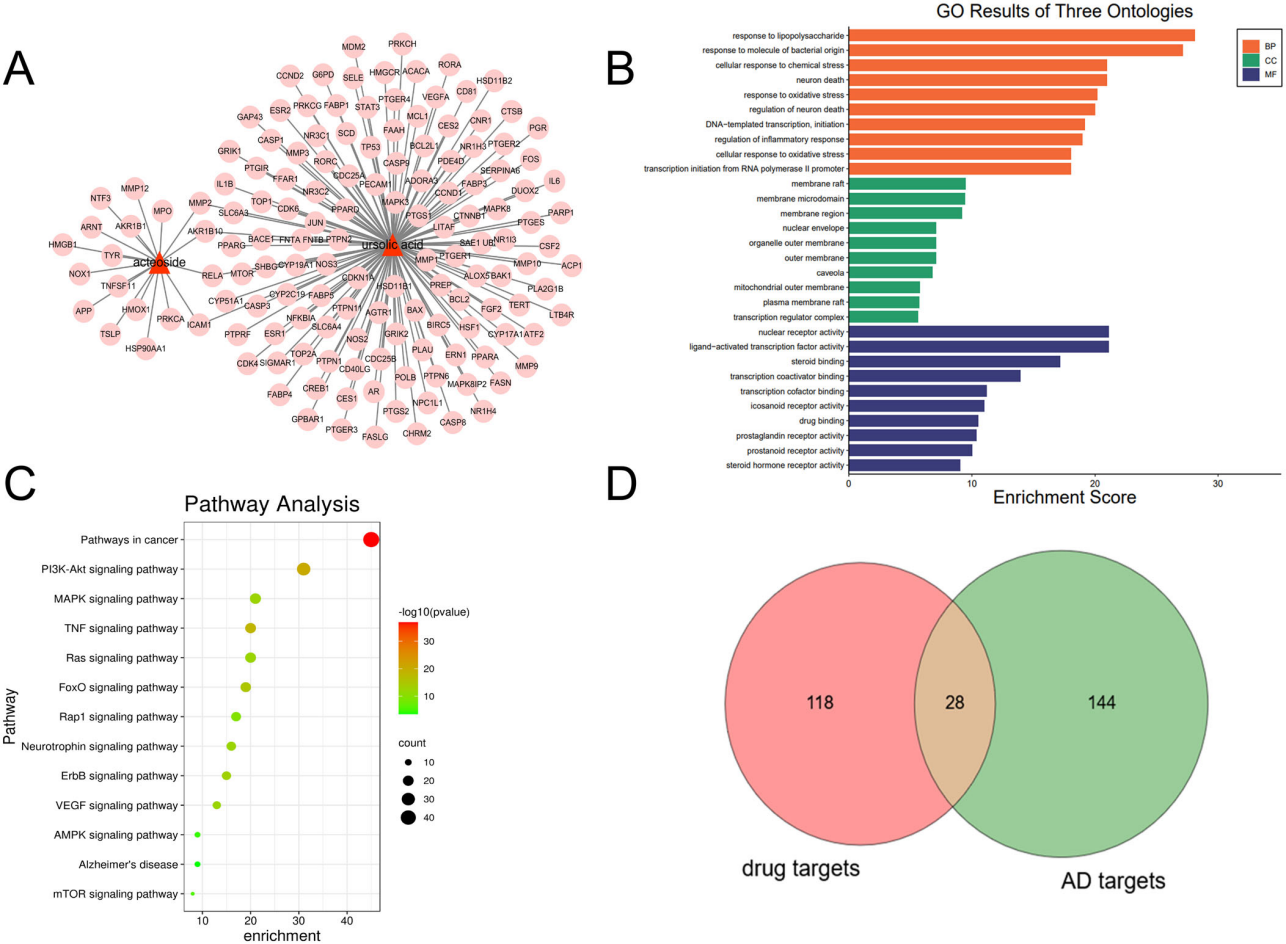
Targets of UA and ATS. A, Potential targets of ATS and UA. B, GO enrichment analysis for targets of UA and ATS. C, KEGG pathway enrichment analysis for targets of UA and ATS (Count number ≥15), *q* value refers to -log10 (*p*-value). D, Shared targets between UA+ATS targets and AD targets.

Through the collection and prediction from TCMSP, STITCH, and SwissTargetPrediction databases, 149 targets corresponding to UA and ATS were found. The corresponding relationship between the compounds and the target is shown in Figure 1(A). GO enrichment analysis showed that these targets mainly exist in membrane raft, membrane microdomain, and other regions, and participate in biological processes such as response to lipopolysaccharide and molecule of bacterial origin, neuron death and response to oxidative stress, and have molecular functions such as nuclear receptor activity, ligand − activated transcription factor activity and steroid binding, etc. (Figure 1(B)). KEGG pathway enrichment analysis indicated 115 pathways affected by UA and ATS (*p* < 0.05). The top 16 pathways included cancer, metabolism, PI3K-Akt, MAPK, TNF, RAS, Fox0, HIF-1, thyroid hormone signalling pathway, focal adhesion, and HTLV-I infection pathways (Figure 1(C)).

### Key targets of UA and ATS acting on AD

**Figure 2.**
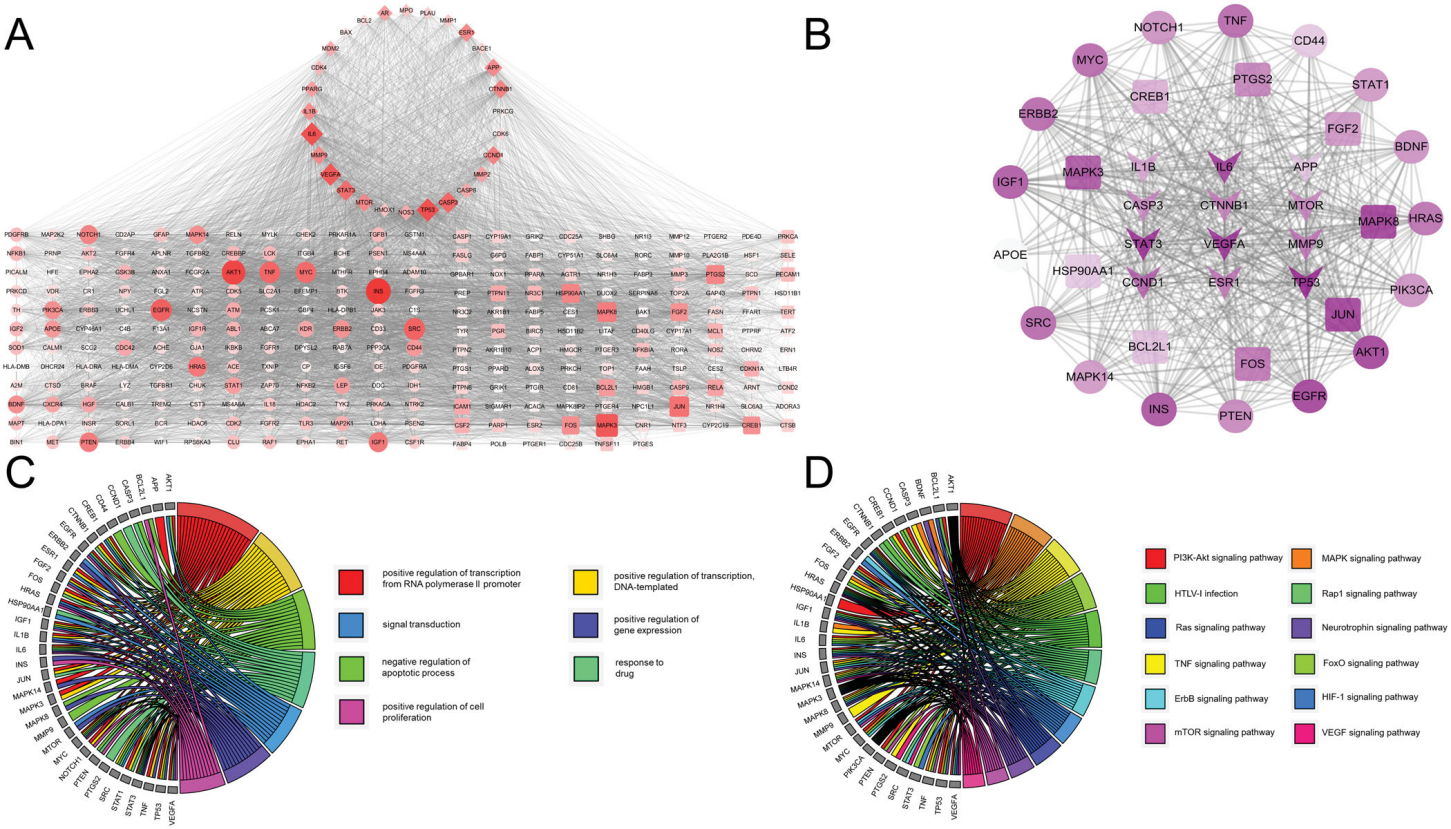
Key targets of UA and ATS for AD treatment. (A) UA + ATS target-AD targets network. Eclipse is the AD-related target, the round tangle is the drug target, and diamond is the shared target. The node size is proportional to the centrality obtained from topology analysis. (B) Network of key targets. Tangle targets are drug targets, round targets are AD targets, and V-shaped targets are shared targets. (C) Biological process enrichment analysis for key targets (Count number >12). (D) KEGG pathway enrichment analysis for key targets (Count number >12).

To determine the relationship between the potential targets for UA + ATS and AD-related targets, we used the Venn online tool (https://bioinfogp.cnb.csic.es/tools/venny/index.html) to obtain 28 shared targets for UA + ATS and AD (Figure 1(D)). Subsequently, we established the interaction network of UA + ATS targets and AD-related targets (Figure 2(A)). The network consists of 288 nodes and 6456 edges. Eclipse is the AD-related target, round tangle is the drug target, and diamond is the shared target. The node size is proportional to the centrality obtained from topology analysis. Thirty-eight key targets were obtained, and an interaction network of key targets was established (Figure 2(B)). The network consists of 38 nodes and 467 edges. Tangle targets are from drug targets, the round targets are from AD targets, and V-shaped targets are shared targets. The information for the top 30 key targets is listed in Table 1.

**Table 1.** Key targets of UA + ATS acting on AD (top 30).

Target	Name	Degree	Betweenness centrality	Closeness centrality
INS	Insulin	193	0.080	0.720
AKT1	AKT serine/threonine kinase 1	178	0.037	0.695
TP53	Tumour protein p53	168	0.037	0.680
IL6	Interleukin 6	167	0.043	0.677
MAPK3	Mitogen-activated protein kinase 3	159	0.025	0.667
VEGFA	Vascular endothelial growth factor A	158	0.024	0.662
EGFR	Epidermal growth factor receptor	150	0.038	0.652
SRC	SRC Proto-Oncogene, Non-Receptor Tyrosine Kinase	148	0.032	0.654
CASP3	Caspase 3	144	0.019	0.646
TNF	Tumour necrosis factor	144	0.028	0.644
MYC	MYC proto-oncogene, bHLH transcription factor	140	0.013	0.639
STAT3	Signal transducer and activator of transcription 3	138	0.015	0.631
JUN	Jun proto-oncogene, AP-1 transcription factor subunit	132	0.009	0.627
HRAS	HRas Proto-Oncogene, GTPase	126	0.013	0.617
IGF1	Insulin like growth factor 1	126	0.010	0.619
MAPK8	Mitogen-activated protein kinase 8	122	0.009	0.614
PTEN	Phosphatase and tensin homolog	122	0.009	0.612
CCND1	Cyclin D1	117	0.010	0.594
NOTCH1	Notch Receptor 1	116	0.007	0.618
CTNNB1	Catenin beta 1	115	0.012	0.604
HSP90AA1	Heat Shock Protein 90 Alpha Family Class A Member 1	114	0.017	0.604
PTGS2	Prostaglandin-Endoperoxide Synthase 2	114	0.014	0.606
MTOR	Mechanistic Target Of Rapamycin Kinase	112	0.007	0.601
APP	Amyloid beta precursor protein	110	0.039	0.607
ESR1	Estrogen Receptor 1	110	0.008	0.598
MMP9	Matrix metallopeptidase 9	110	0.006	0.600
FOS	Fos proto-oncogene, AP-1 transcription factor subunit	106	0.005	0.594
PIK3CA	Phosphatidylinositol-4,5-Bisphosphate 3-Kinase Catalytic Subunit Alpha	105	0.011	0.593
IL1B	Interleukin 1 Beta	105	0.011	0.593
MAPK14	Mitogen-activated protein kinase 14	105	0.008	0.595

To clarify the characteristics of key targets at the molecular functional level, biological process and KEGG enrichment analysis were carried out, and chord charts were drawn for those with count number > 12 (Figure 2(C,D)). These targets mainly exist in the nucleus, cytoplasm, plasma membrane, nucleoplasm, and other regions, participate in biological processes such as transcriptional regulation, drug reaction, signal transduction, apoptosis, and have molecular functions such as binding proteins and enzymes. KEGG pathway enrichment analysis demonstrated that 106 pathways were affected by key targets (*p* < 0.05). Among them, the pathways of count number > 12 include cancer, PI3K-Akt, MAPK, TNF, FOXO, thyroid hormone signalling pathway, and local adhesion pathways.

### Binding capacity between UA + ATS and key targets by molecular docking

**Figure 3.**
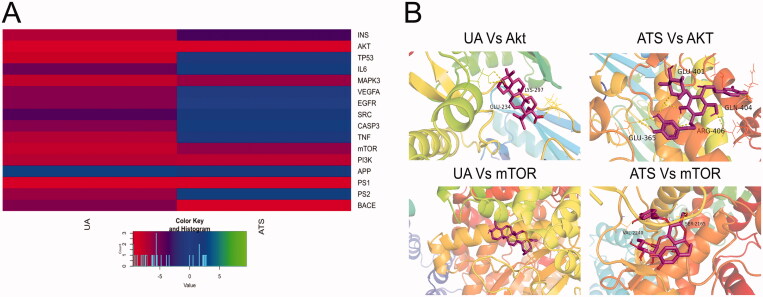
Molecular docking. (A) The heat map of UA and ATS with the top key target docking score. The closer to red, the lower the binding energy, and the more stable the structure are. (B) The molecular docking visualization results of the compounds binding to AKT and mTOR.

We performed molecular docking between UA and ATS and key targets, and the docking results are shown in Figure 3(A). The binding energy between the compound and the target is less than 0, indicating that the ligand and the receptor can spontaneously bind. The smaller the binding energy, the stronger the binding force and more stable the structure. It is worth noting that UA and ATS had strong binding properties with Akt, mTOR, PI3K, and MAPK3, and the binding energies were all less than −5 kcal/mol. UA Vs. Akt, UA Vs. mTOR, ATS Vs. Akt, ATS Vs. mTOR showed good performances of binding, and their visualization results were exhibited in Figure 3(B). The binding energies of UA Vs. Akt are −9.46 kcal/mol, and they combine to form a hydrophobic structure and two hydrogen bonds. The hydrogen bond residues are Lys297 and Glu34. The binding energies of UA Vs. mTOR are −7.81, with no hydrogen bonds. The binding energies of ATS Vs. Akt are −9.5 kcal/mol, and they combine to form a hydrophobic structure and 4 hydrogen bonds. The hydrogen bond residues are Glu365, Glu401, Arg406, and Gln404. The binding energies of ATS Vs. mTOR are −6 kcal/mol, and they combine to form a hydrophobic structure and four hydrogen bonds. The hydrogen bond residues are Val2240 and Ser2165.

### UA and ATS protects H_2_O_2_-induced nerve damage

**Figure 4.**
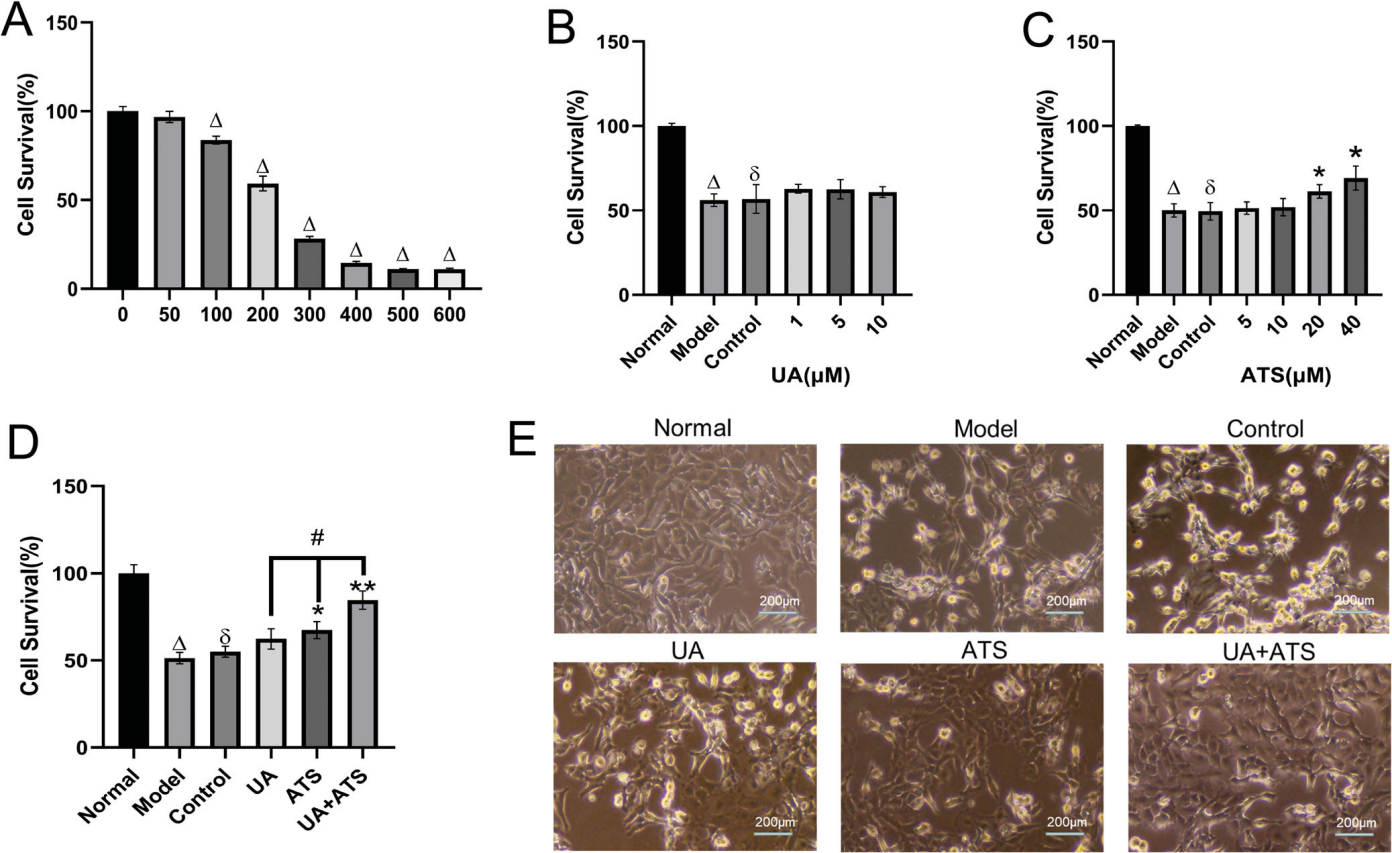
Effects of UA and ATS on H_2_O_2_ -induced nerve damage. (A) Effects of H_2_O_2_ on PC12 cells viability. (B) PC12 cells were treated with different concentrations of ATS for 24 h, then treated with 250 μM H_2_O_2_ for 4 h, and cell viability was evaluated by CCK-8 assay. (C) PC12 cells were treated with different concentrations of UA for 24 h, then treated with 250 μM H_2_O_2_ for 4 h, and cell viability was evaluated by CCK-8 assay. (D) PC12 cells were pre-incubated with UA (5 μM) or, and ATS (40 μM) for 24 h, then treated with 250 μM H_2_O_2_ for 4 h, and cell viability were evaluated by CCK-8 assay. E, Cell morphology of each group under a light microscope (×200). *p < 0.05, **p < 0.01, vs. control; ^Δ^*p* < 0.01 vs. normal; ^ð^*p* > 0.05 vs. model; ^#^*p* < 0.05 vs. UA or ATS group.

The network pharmacological results revealed that UA and ATS played an anti-AD role by participating in biological processes such as transcriptional regulation, signal transduction, and apoptosis. We further observed the neuroprotective effect of UA and ATS in H_2_O_2_-induced nerve damage in PC12 cells. The results showed that 100–600 μM H_2_O_2_ treatment for 4 h significantly inhibited PC12 cells viability compared with the control group (*p* < 0.05) (Figure 4(A)). We selected H_2_O_2_ with a half inhibition value (IC_50_ value) of 250 μM to establish oxidative damage for subsequent experiments.

Pre-incubated with UA (1–10 μM) and ATS (5–40 μM) significantly increased cell viability compared with the control group (*p* < 0.05), which implied that UA and ATS produced a protective effect in H_2_O_2_-induced nerve damage (Figure 4(B,C)). The ED_50_ value of UA (5 μM) and ATS (40 μM) were employed for subsequent experiments. The combination of UA and ATS showed higher protective effects than that of the UA and ATS single treatment alone (*p* < 0.05) (Figure 4(D)). The combination index [Bliss independence model (Foucquier and Guedj 2015)] of UA and ATS was 0.64 (CI = (E_A_+E_B_-E_A_E_B_)/E_AB_, E value was taken as the effective value, which is the degree of protection compared to the control group in this study. CI < 1 indicated a more significant effect than two drugs used alone, suggesting UA and ATS showed a synergistic effect.

We also observed the cell morphology of each group under a light microscope. The PC12 cells in the normal group were uniform in size, polygonal and fusiform in shape, packed in body, clear in outline, with a small protrusion on edge, strong refraction, and close adherence to the wall. The number of normal-shaped cells in the model and control group was significantly reduced, and the cells were different in size and round in shape. The cells were swollen and shrivelled, the edges of the cells were rough, the adherent cells were relaxed, and the refractive index was poor – nearly half of the cells detached from the wall due to death. In the UA and ATS groups, the cell morphology was improved, some cells showed polygons, and the refractive rate increased, and the number of normal cells was increased. Especially in the UA + ATS group, the number of normal cells was raised and the cell morphology and refractive rate were not significantly different from that of the normal group (Figure 4(E)).

### UA and ATS protects H_2_O_2_-induced apoptosis

**Figure 5.**
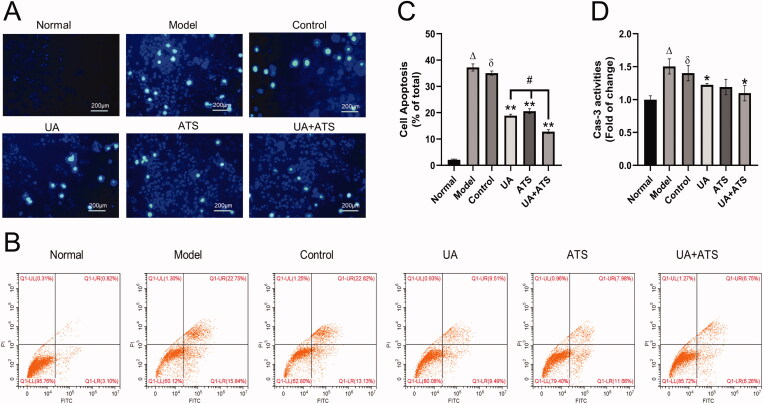
Effects of UA and ATS on H_2_O_2_-induced apoptosis. PC12 cells were treated with ATS and/or UA for 24 h, then treated with 250 μM H_2_O_2_ for 4 h, and subjected to Hoechst 33258 staining (A, ×200), apoptosis detection by flow cytometry (B and C), caspase-3 activates detection (D), and *p < 0.05, **p < 0.01, vs. control; ^Δ^*p* < 0.01 vs. normal; ^ð^*p* > 0.05 vs. model; ^#^*p* < 0.05 vs. UA or ATS group.

Subsequently, we observed the effects of UA and ATS on apoptosis. The results of Hoechst 33258 staining are shown in Figure 5(A). The cells in the normal group showed weak fluorescence staining. In contrast, the cells in the model group and the control group treated with H_2_O_2_ could absorb Hoechst 33258 and showed strong blue fluorescence, and dense blue fluorescent particles could be seen in the nucleus or cytoplasm. Apoptotic nuclear morphology includes chromatin agglomeration, marginalization, and lysis of the cell nucleus. These defects could be saved by pre-treatment with UA, ATS, and UA + ATS, thus reducing the proportion of apoptotic cells, and the effect of the UA + ATS group was the most significant. The results of Annexin V-FITC/PI double staining and flow cytometry analysis revealed that PC12 cells apoptosis was provoked by H_2_O_2_. At the same time, pre-treatment with UA, ATS, and UA + ATS could diminish the apoptosis induced by H_2_O_2_ (*p* < 0.01), and the effects of UA + ATS were more potent than either treatment alone (*p* < 0.05) (Figure 5(B,C)).

### UA and ATS inhibits caspase-3 and alleviate down-regulation of MMP

Caspase family protease plays a significant role in the process of cell apoptosis. Among the family members, caspase-3 is the executive protease of cell apoptosis, so it is closely correlated with cell apoptosis (Suzuki et al. 1997). Additionally, caspase-3 ranks high among the key targets derived from network pharmacology above. In this study, we observed that both UA and ATS alone or in combination could inhibit H_2_O_2_-activated caspase-3 in PC12 cells (*p* < 0.05), and the UA + ATS group showed the best effect (Figure 5(D)).

**Figure 6.**
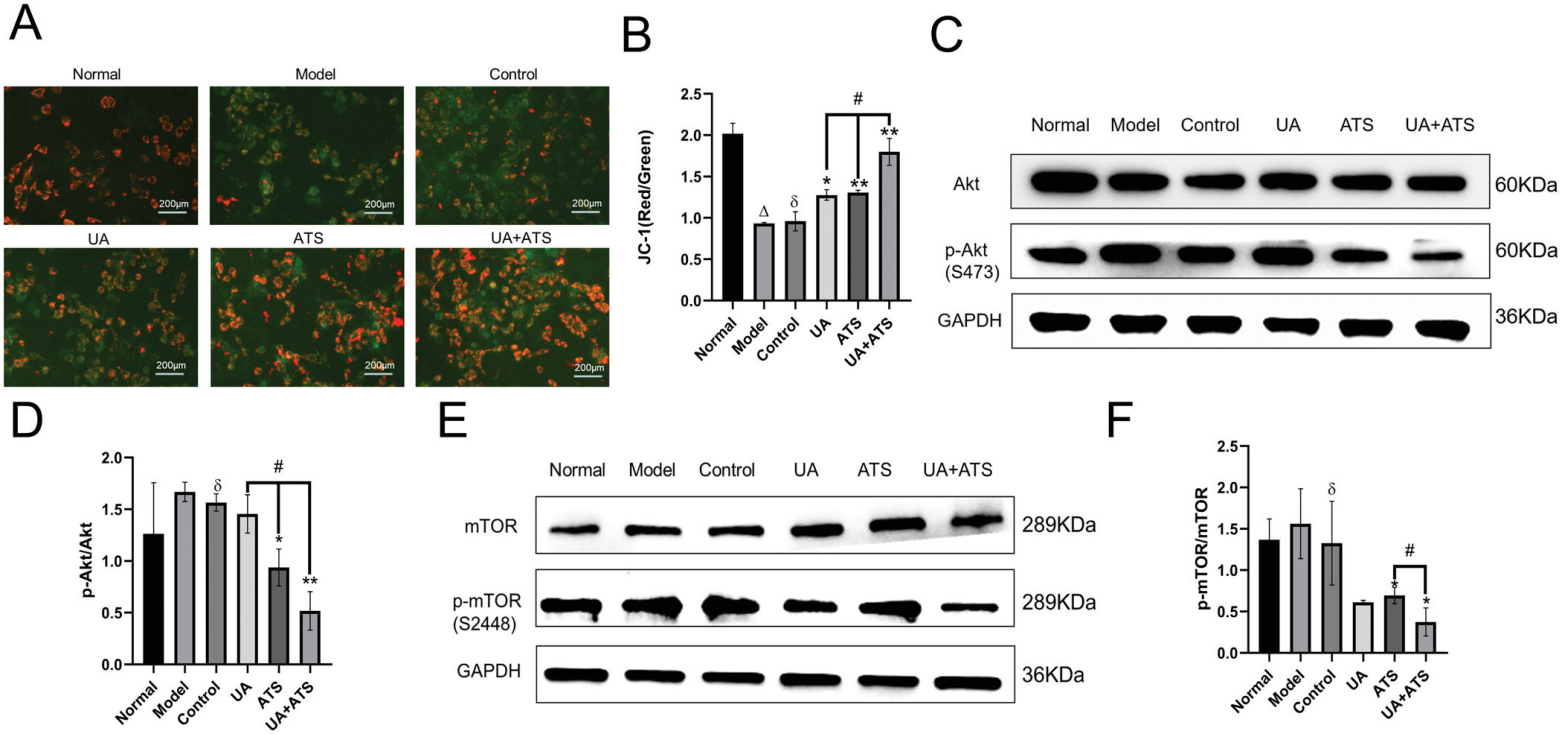
Effects of UA and ATS on H_2_O_2_-activated proteins phosphorylation and MMP. MMP detection by JC-1 staining (A (×200) and B). PC12 cells were treated with ATS and/or UA for 24 h, then treated with 250 μM H_2_O_2_ for 4 h, and subjected to Western blot with indicated antibodies (C and E) and quantified by Image J (D and F). *p < 0.05, **p < 0.01, vs. control; ^Δ^*p* < 0.01 vs. normal; ^ð^*p* > 0.05 vs. model; ^#^*p* < 0.05 vs. UA or ATS group.

Mitochondria play a central role in energy production, which is necessary for almost all cellular activity. Notably, they are vital in neurons that require a lot of ATP. Therefore, damaged mitochondria lead to pathological state, and mitochondrial dysfunction is one of the earliest and most prominent features of AD (Wang et al. 2019). Mitochondria is related to caspase-9 and is followed by caspase-3 activation (Obulesu and Lakshmi 2014). In this study, we detected MMP by JC-1 staining. When MMP was normal, JC-1 formed J-aggregates and produced red fluorescence in the cytoplasm. However, if cells were damaged and MMP decreased, JC-1 became a monomer and produced green fluorescence. As shown in Figure 6(A,B), we found that UA and ATS alone or in combination could reduce MMP down-regulation in PC12 cells after H_2_O_2_ treatment. The combination of UA and ATS showed a higher effect than either treatment alone (*p* < 0.05).

### UA + ATS regulates the Akt/mTOR signalling pathway

KEGG pathway enrichment showed that UA and ATS could regulate Akt and mTOR pathways and bind to Akt and mTOR. We further detected the expression and phosphorylation of Akt and mTOR by Western blot. As shown in Figure 6(C–F), H_2_O_2_ up-regulated the phosphorylation of Akt and mTOR, UA and ATS alone or in combination could lessen the up-regulated phosphorylation of Akt and mTOR in PC12 cells after H_2_O_2_ intervention (*p* < 0.01), and the UA + ATS group exhibited the best effect on the phosphorylation of Akt (*p* < 0.05).

### Changes in cell viability after rapamycin stimulation of autophagy

**Figure 7.**
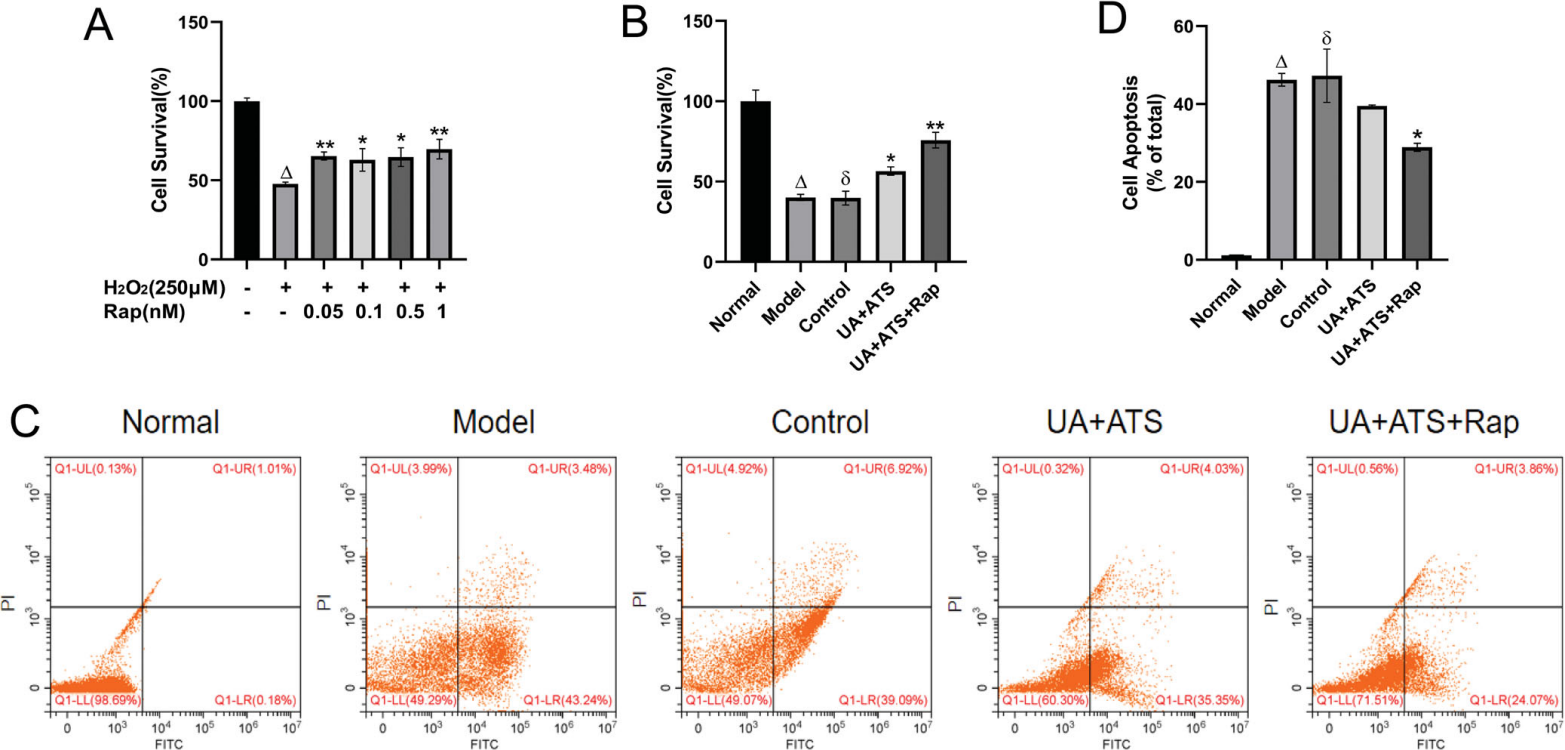
Changes in cell viability after Rapamycin stimulation of autophagy. (A) the reasonable concentration of rapamycin acting on PC12 cells. (B) CCK-8 cell viability assay. (C and D) Annexin-V FITC PI double staining cell apoptosis assay. *p < 0.05, **p < 0.01, vs. model (A), control (B and C); ^Δ^*p* < 0.01 vs. normal; ^ð^*p* > 0.05 vs. model (B and C).

To address the role of UA + ATS in the Akt/mTOR pathway, we added a positive control group. Firstly, we used the CCK-8 method to explore the reasonable concentration of rapamycin (Rap, mTOR inhibitor, and autophagy agonist) acting on PC12 cells. Subsequently, CCK-8 cell viability assay and Annexin-V FITC PI double staining cell apoptosis assay were performed. Rap was used to intervene in H_2_O_2_-treated PC12 cells, and it was found that cell viabilities were increased while the apoptosis of PC12 cells were decreased after the autophagy excitation induced by Rap (Figure 7) (*p* < 0.05). UA + ATS improved the apoptosis caused by H_2_O_2_, which had the same trend with the improvement of cell apoptosis after the autophagy excitation induced by Rap (Figure 7).

### UA + ATS regulates ATG5 and Beclin-1 through the Akt/mTOR signalling pathway

**Figure 8.**
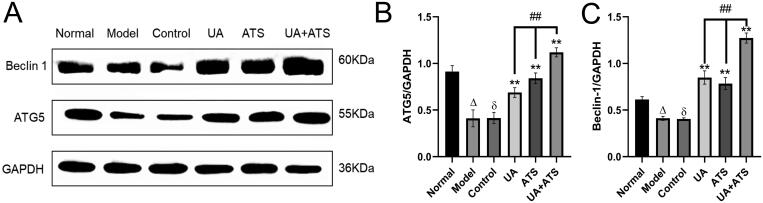
Effects of UA and ATS on regulating ATG5 and Beclin-1 (A) and quantified by Image J (B and C) *p < 0.05, **p < 0.01, vs. control; ^Δ^*p* < 0.01 vs. normal; ^ð^*p* > 0.05 vs. model; ^#^*p* < 0.05 vs. UA or ATS group.

ATG5 is an essential marker of autophagosome formation, while Beclin-1 is the core component of the PI3K-III complex, which aggregates a variety of autophagic proteins during the formation of autophagosomes. To explore the specific mechanism of UA + ATS regulating autophagy through the Akt/mTOR pathway, we detected the expression of ATG5 and Beclin-1. As shown in Figure 8, H_2_O_2_ down-regulated the expression of ATG5 and Beclin-1. UA and ATS alone or in combination increased the expression of ATG5 and Beclin-1 in PC12 cells after H_2_O_2_ intervention (*p* < 0.01), and the UA + ATS group had the best effect (*p* < 0.01).

## Discussion

AD is a global health problem that leads to a reduced quality of life and a severe burden on patients. UA and ATS are the essential active components of *C. officinalis* Sieb. et Zucc. (Cornaceae). and *R. glutinosa* (Gaetn.) Libosch. ex Fisch. et Mey. (Scrophulariaceae), respectively. UA can exert neuroprotective effects by restraining ROS and simultaneously enhancing antioxidant enzyme activity (Habtemariam 2019), relegating Aβ neurotoxicity and inflammatory response (Yoon et al. 2014), and intensifying the expression of brain growth-related protein GAP43 (Lu et al. 2007). ATS can yield neuroprotective effects by recuperating Aβ deposition in the brain of AD animal models (Shiao et al. 2017), transferring Nrf2 into the nucleus to up-regulate the expression of downstream antioxidant enzymes (Wang et al. 2012), regulating immune function and autophagy, and other pathways. In the present study, we identified the key targets of UA and ATS acting on AD through network pharmacology firstly. We established a target-pathway interaction network to explore the potential mechanisms further. Successively, we demonstrated that UA and ATS can hinder the apoptosis of PC12 cells induced by H_2_O_2_, and the mechanism may be associated with the regulation of the Akt/mTOR signalling pathway.

In the present study, 38 key targets of UA and ATS were identified for AD treatment. KEGG pathway analysis of 38 key targets showed that UA and ATS could regulate multiple pathways, including PI3K, Akt, and mTOR, so we focussed on the PI3K/Akt/mTOR signalling pathway. PI3K is an essential member of the phospholipid kinase family. Akt, also known as protein kinase B (PKB), is an important downstream target kinase of PI3K signalling. mTOR is also a member of the protein kinase family, and is a downstream effector protein of the PI3K/Akt signalling pathway, whose substrate regulation is related to the synthesis of proteins related to cell growth, survival, and proliferation, as well as the regulation of autophagy. Recent studies have revealed that mTOR activation may be responsible for the breakdown of the blood-brain barrier in AD, the hyperphosphorylation of tau protein, and the persistent metabolic dysfunction associated with the formation of senile plaques (Stanciu et al. 2021). mTOR drives cerebrovascular dysfunction in AD model by downgrading the activity of endothelial nitric oxide synthase (eNOS), while rapamycin restores eNOS-dependent cerebrovascular function by hindering the activity of mTOR (Van Skike et al. 2021). Apart from this, mTOR also mediated central insulin dysfunction in the AD model mice. The cognitive ability, Aβ, and Tau levels of AD model mice were all improved after mTOR deletion. A large number of studies have exposed that decreasing the activation of PI3K/Akt/mTOR signals at multiple levels can alleviate ageing to a certain extent and prolong the healthy life span of organisms (Caccamo et al. 2013; O’Neill 2013). Therefore, reducing the PI3K/Akt/mTOR signalling pathway plays a crucial role in inhibiting the development of AD pathology and the decline of cognitive ability.

Consistent with the above findings, our experiment confirmed that UA + ATS could inhibit the apoptosis induced by H_2_O_2_ in PC12 cells. We found that the combination of UA and ATS could cut down the upregulation of phosphorylation of Akt and mTOR in PC12 cells after H_2_O_2_ treatment, which indicated that the phenotypic results achieved by UA + ATS such as anti-apoptosis, restoration of mitochondrial membrane potential, and reduction of caspase3 release might be performed by restraining the Akt/mTOR signalling. The mTOR mediated signalling pathway is the classical regulatory pathway of autophagy. Under the condition of adequate nutrition, growth factors, glucose, amino acids, and other signals can directly interact with or phosphorylate autophagy-related factor serine/threonine kinase 1 (ULK1) through mTOR complex 1 (mTORC1) of the mTOR signalling pathway, affecting the formation of ULK complex and forming negative regulation of autophagy. Under starvation or oxidative stress, mTORC1 is constrained, and ULK1 is motivated to form ULK complex, which is transferred from the cytosol to autophagy prosomes, thus initiating the process of autophagy (King et al. 2021). The loss of ATG5, a key gene essential for autophagosome maturation, led to a significant increase in the number and size of Aβ plaques in the brains of mice (Komatsu et al. 2006). A PD-related study showed that depletion of ATG5 in microglia exacerbated neuroinflammation and loss of dopaminergic neurons in the substantia nigra in α-Syn overexpressing mice (Tu et al. 2021). It can be seen that the loss or low expression of ATG5 aggravates the deposition of pathological products of neurodegeneration, and ATG5 has a certain neuroprotective effect. Beclin-1 reflects the level of autophagy induction. Studies have shown that the expression of Beclin-1 is reduced in the brain of AD. Beclin-1 loss has been found to stimulate the deposition of Aβ in AD cell models and animal models, and the loss Beclin-1 impels autophagy, thereby promoting the pathological changes of AD (Salminen et al. 2013).

In this study, we have proved that UA + ATS can regulate the expression of autophagy-related proteins ATG5 and Beclin-1, thus affecting autophagy. Consequently, our next research will focus on exploring the regulatory effect of UA + ATS on ULK1 and the relationship between the regulation of UA + ATS on autophagy and oxidative stress in the AD cell models.

Autophagy and apoptosis are two complex processes closely related to each other to maintain cell homeostasis. At present, the relationship between autophagy and apoptosis is a hot topic and controversial. Several studies have shown that autophagy can reduce apoptosis. Autophagy can block apoptosis by inhibiting mitochondrial pathways and the activity of caspase, while autophagy is capable of accelerating pathologically ill-treated cells apoptosis (Prerna and Dubey 2022). On the contrary, the occurrence of autophagy will be intensified if apoptosis is banned (Marino et al. 2014). Amir et al. (2013) found that the inhibition of autophagy by liver-specific ATG-7 knockout increased caspase-8 activity and apoptosis. Linnemann et al. (2017) found that interleukin 6 protects pancreatic β-cells from apoptosis by inhibiting of mTOR complex 1 and then stimulating autophagy；the study Zhou et al. (2021) showed that autophagy can alleviate cadmide-induced apoptosis of mouse testicular germ cells. Autophagy can also alleviate cell death by selectively reducing the abundance of pro-apoptotic proteins in cytosol. In addition, the activation of apoptosis-related proteins can also inhibit autophagy by degrading autophagy related proteins such as Beclin-1, ATG-4D, ATG-3 and ATG-5. It is worth noting that several studies have suggested that autophagy may promote apoptosis. In neurodegenerative diseases represented by AD, inhibition of autophagy and increased apoptosis were observed. Combined with the results of this study, we support the idea that autophagy can reduce apoptosis.

In addition to the above pathways, INS, TP53, IL6, MAPK, and VEGFA, which rank among the top key targets, are also closely related to AD. The latest studies found that insulin transduction affects the function of neurotransmitters by adjusting glucose energy metabolism, and plays a crucial part in the cognition and synaptic plasticity of neurons. Insulin resistance leads to impaired insulin signals in the AD brain, and eventually makes the formation of neurofibrillary entanglement possible (Sharma and Singh 2020; Kshirsagar et al. 2021). The transcription factor p53 is known for its role in tumour suppression, and recent studies have shown that dysregulation of p53 activity may initiate various peripheral and brain changes in early AD (Abate et al. 2020). In the presence of DNA damage, phosphorylated p53 is localized outside the nucleus, and p53-mediated DNA damage responders are significantly decreased in the AD brain (Farmer et al. 2020). It is well known that neuroinflammation is one of the necessary pathological features of AD, and IL6 is a pleiotropic inflammatory cytokine, which plays a vital role in the acute inflammatory response and the regulation of neuroimmune response. Clinical studies have revealed that IL-6 is associated with the aggregation of Aβ and the presence of highly phosphorylated Tau protein in the AD brain (Chen et al. 2012).

MAPKs play a crucial role in the management of cell response to the environment, and contribute to gene expression, cell growth, and apoptosis regulation, and the activation of the p38MAPK signalling pathway is closely related to AD. Recent studies have disclosed that p38 and JNK in MAPKs are involved in Aβ-induced astrocyte proliferation and influence AD by mediating the motivation of astrocytes (Saha et al. 2020). VEGF is a critical factor that promotes angiogenesis and has a protective effect on the nervous system. Studies have shown that VEGF has a protective effect on cognitive function in AD models by improving mitochondrial dysfunction (Liu et al. 2021) and escalating mitochondrial number, and shrinking Aβ production and deposition (Guo et al. 2019). These essential factors have not been verified by cell experiments in this study, so we will consider conducting subsequent experiments.

## Conclusions

The present study demonstrated that the neuroprotective of UA and ATS was related to inhibiting the phosphorylation of Akt and mTOR for restoring mitochondrial membrane potential, reducing the activation of caspase-3, and increasing the expression of ATG5 and Beclin-1, thus reducing cell apoptosis. In this study, the key targets and signal pathways of UA and ATS in the treatment of AD were identified from the perspective of network pharmacology. The Akt/mTOR signalling pathway was verified by molecular docking and cell experiments, which has guiding significance for the application of UA and ATS and provides new clues for future research and development of anti-AD drugs.

## Author contributions

HMA and BH designed the study, coordinated technical support and funding; BH revised the manuscript; YJQ wrote the main text; MRD, CG, LMZ, RRZ, and JFC retrieved and organized documents. All authors read and approved the final manuscript.

## Data Availability

The datasets used and/or analysed during the current study are available from the corresponding author on reasonable request.
